# The Endosymbiotic Bacterium *Wolbachia* Induces Resistance to Dengue Virus in *Aedes aegypti*

**DOI:** 10.1371/journal.ppat.1000833

**Published:** 2010-04-01

**Authors:** Guowu Bian, Yao Xu, Peng Lu, Yan Xie, Zhiyong Xi

**Affiliations:** 1 Department of Entomology and Genetics Program, Michigan State University, East Lansing, Michigan, United States of America; 2 Center for Statistical Training & Consulting, Michigan State University, East Lansing, Michigan, United States of America; Stanford University, United States of America

## Abstract

Genetic strategies that reduce or block pathogen transmission by mosquitoes have been proposed as a means of augmenting current control measures to reduce the growing burden of vector-borne diseases. The endosymbiotic bacterium *Wolbachia* has long been promoted as a potential vehicle for introducing disease-resistance genes into mosquitoes, thereby making them refractory to the human pathogens they transmit. Given the large overlap in tissue distribution and intracellular localization between *Wolbachia* and dengue virus in mosquitoes, we conducted experiments to characterize their interactions. Our results show that *Wolbachia* inhibits viral replication and dissemination in the main dengue vector, *Aedes aegypti*. Moreover, the virus transmission potential of *Wolbachia*-infected *Ae. aegypti* was significantly diminished when compared to wild-type mosquitoes that did not harbor *Wolbachia*. At 14 days post-infection, *Wolbachia* completely blocked dengue transmission in at least 37.5% of *Ae. aegypti* mosquitoes. We also observed that this *Wolbachia*-mediated viral interference was associated with an elevated basal immunity and increased longevity in the mosquitoes. These results underscore the potential usefulness of *Wolbachia*-based control strategies for population replacement.

## Author Summary

Dengue fever and its associated condition, dengue hemorrhagic fever, are emerging globally as the most important arboviral diseases currently threatening human populations. Dengue virus is transmitted to humans by aedine mosquitoes, primarily *Aedes aegypti* and, to a lesser extent, *Aedes albopictus*. No treatment or vaccine is currently available for dengue fever; thus, vector control remains the primary intervention tool. One novel control strategy for reducing or blocking dengue transmission by mosquitoes involves the endosymbiotic bacterium *Wolbachia*, which has long been promoted as a potential vehicle for introducing anti-dengue genes into mosquitoes. Here, we have characterized the interaction of *Wolbachia* with dengue viruses in *Ae. aegypti*. *Wolbachia* alone was able to inhibit viral replication, dissemination and transmission in these mosquitoes. In addition, this *Wolbachia*-mediated viral interference was associated with an elevation of basal immunity and increase in longevity in the mosquitoes. Our study provides novel insights into the usefulness of *Wolbachia* for blocking dengue transmission by mosquitoes.

## Introduction

Dengue fever and its associated condition, the highly lethal dengue hemorrhagic fever, are emerging globally as the most important arboviral diseases currently threatening human populations. Approximately 2.5 billion people are at risk of contracting dengue-associated disease, with an estimated 50–100 million cases occurring each year [1]. Dengue virus (DENV) is transmitted to humans by aedine mosquitoes, primarily *Aedes aegypti* and, to a lesser extent, *Aedes albopictus*. At present, no treatment or vaccine is available for dengue fever; thus, vector control is currently the primary intervention tool. One such method is population replacement, in which natural *Ae. aegypti* populations would be replaced with modified populations that are unable to transmit DENV. Recently, significant progress has been made in producing *Ae. aegypti* strains that are refractory to DENV [2],[3] and in exploring transgene drivers for population replacement [4],[5].

One of the most promising transgene drivers is a maternally transmitted Gram-negative endosymbiotic bacterium, *Wolbachia*. Significantly, *Wolbachia* is able to spread rapidly within an uninfected *Ae. aegypti* laboratory population after the population has been seeded with infected females [6]. *Wolbachia* induces a reproductive abnormality known as cytoplasmic incompatibility (CI), which results in early embryo death when a *Wolbachia*-infected male has mated with a female that is uninfected or harboring a different *Wolbachia* type. Since uninfected males can successfully mate with infected females, *Wolbachia* and any gene it carries can spread quickly in a population.

Two control approaches using *Wolbachia*-based population replacement have been proposed: One potential approach involves linking a transgene to *Wolbachia*. The mosquito's vectorial capacity is reduced as the transgene is carried by *Wolbachia* into the target population. The second approach utilizes the ability of *Wolbachia* itself to modify both the sexual reproduction and vectorial capacity of the host. For example, after mosquitoes are fed an infectious blood meal, a period of 7–14 days (depending on environmental and intrinsic factors) is required before the mosquitoes are able to transmit DENV to a new host [7],[8]. One strain of *Wolbachia*, called *popcorn*, has been shown to reduce the longevity of mosquitoes, causing the insects to die before they can transmit viruses [9],[10]. *Wolbachia*, introduced into wild vector populations, will inevitably encounter DENV, given large overlap in tissue distribution and intracellular localization of these two microorganism [8],[11]. However, at present it is still unclear how *Wolbachia* and DENV interact in the mosquito.

Recent studies in *Drosophila* have shown that *Wolbachia* can confer resistance to diverse RNA viruses and protect flies from virus-induced mortality [12],[13]. Two groups have independently reported that *Wolbachia* significantly reduces the infection level of the *Drosophila* C virus, a member of the *Dicistroviridae*, in different lines of *D. melanogaster*. When compared to flies cured of *Wolbachia* infection, those with an active *Wolbachia* infection showed a significantly delayed mortality induced by *Drosophila* C virus infection. A similar antiviral effect was also observed in *Wolbachia*-infected flies challenged with cricket paralysis virus (*Dicistroviridae*), Nora virus (a new picorna-like virus family) and flock house virus (*Nodaviridae*). Since *Drosophila* C virus and DENV are both single-stranded positive-sense RNA viruses, these findings strongly support the existence of an overarching mechanism that is also applicable to *Wolbachia*-DENV interactions in their mosquito hosts.

Although *Ae. aegypti* is not naturally infected by *Wolbachia*, infection has been achieved by transfection with *w*AlbB, an infection type of *Wolbachia* from *Ae. albopictus*, by means of embryonic microinjection [6]. *w*AlbB is able to induce a complete CI in *Ae. aegypti*, and this phenotype can be reversed by tetracycline treatment. Moreover, it has a 100% maternal transmission rate, with no fitness costs observed [6]. Since it was introduced, this infection has been stably maintained in *Ae. aegypti* for about 6 years in the laboratory.

Our previous studies have shown that the mosquito's endogenous bacterial flora boost the basal level of immunity in the mosquito and that their removal leads to an increase in the level of dengue infection [14]. In the *Drosophila* S2 cell line, we also found that *Wolbachia* activates immune signaling pathways and induces the expression of antimicrobial peptide genes [15]. In *Ae. egypti*, we have further demonstrated that the anti-bacterial defense responses can control dengue infection [14]. Here, we have asked whether *Wolbachia*, a component of the mosquito's microbial flora, can suppress DENV in *Ae. aegypti*. We found that *Wolbachia* can indeed inhibit DENV infection in *Ae. Aegypt*, and this inhibition was associated with an elevated immune response and an increase in the mosquito's longevity.

## Results

### *Wolbachia*-induced suppression of DENV replication in the mosquito midgut

To characterize the effect of *Wolbachia* on DENV infection in *Ae. aegypti*, we compared DENV dynamics within the mosquito between the original wild-type Waco strain (*Wolbachia*-free) and the Waco-derived WB1 strain (*Wolbachia*-transfected). Mosquitoes were fed sheep blood containing the New Guinea C strain of DENV serotype 2 (DENV-2), with viral titers of 2.0×10^7^ PFU/ml. Using an indirect fluorescent antibody assay (IFA) performed on head squashes at 14 days after the infectious meal, we had previously confirmed that this virus titer resulted in a >90% infection rate in experimentally infected Waco females.

**Figure 1:**
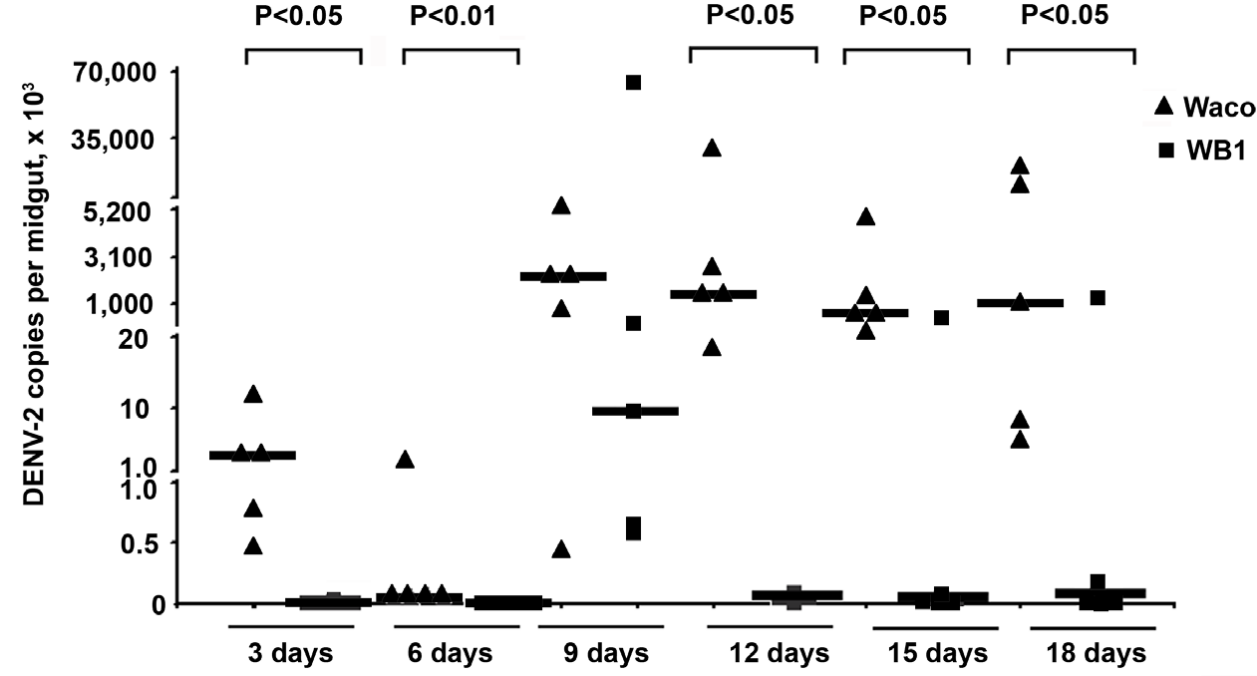
Inhibition of dengue infection in the mosquito midgut by *Wolbachia*. At 3, 6, 9, 12, 15 and 18 days after a blood meal containing DENV-2, mosquito midguts were collected, and the number of genome copies of the DENV genome was determined by qRT-PCR using primers for the NS5 gene; the results were normalized to the *Ae. aegypti* ribosomal protein S7 (RPS6). Lines indicate the median of the five biological replicates. Significance was determined using a Mann-Whitney U test (P<0.05).

The number of copies of DENV-2 genomic RNA was monitored in mosquito midguts by quantitative reverse transcriptase PCR (qRT-PCR) at 3, 6, 9, 12, 15 and 18 days post-infection. Five biological replicates were used for each time point. The number of copies of DENV-2 RNA in the WB1 strain was significantly lower than that in Waco strain at all five time points, with the exception of Day 9 (Mann-Whitney U test, P<0.05) (Figure 1). On Days 3 and 6, DENV was detectable in only one of ten WB1 samples, whereas all the Waco samples were positive for dengue infection. On Day 9, the median viral titer in the WB1 strain reached 9.1×10^3^ genome copies per midgut, whereas the median titer in the Waco strain was 2.4×10^6^ genome copies per midgut; however, this difference was not statistically significant. RNA copy numbers remained at a high level in the midguts of Waco strain mosquitoes from Day 9 to 18.

The strongest *Wolbachia*-mediated virus inhibition was observed on Day 12, when the amount of DENV-2 in the midguts of the WB1 strain mosquitoes was 5.6×10^4^ times lower than that in the midguts of the Waco strain. The dengue infection level in the WB1 midgut was maintained at 1.9×10^4^ and 3.1×10^4^ times lower than that in Waco midguts on Days 15 and 18, respectively. A low level of DENV-2 infection, ranging from 23.0 to 31.5 copies per midgut, was maintained in the midguts of the WB1 line from Day 12 to 18 (Figure 1).

### *Wolbachia*-induced suppression of DENV dissemination to the thorax and head of the mosquito

**Figure 2:**
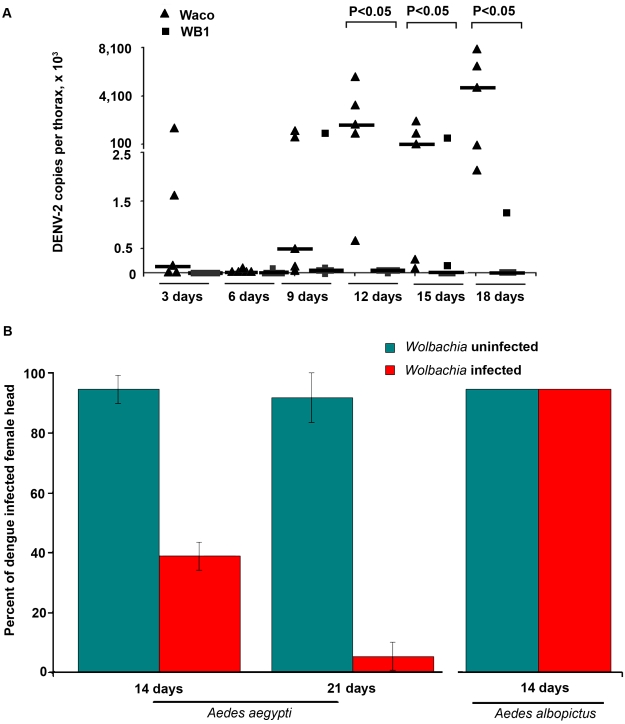
Inhibition of dengue dissemination to the mosquito thorax and head by *Wolbachia*. **A**. At 3, 6, 9, 12, 15 and 18 days after a blood meal containing DENV-2, mosquito thoraces were collected, and genome copies of dengue virus were measured by qRT-PCR using primers for the NS5 gene and normalized with *Ae. aegypti* RPS6. Lines indicate the median of the five biological replicates. Significance was determined using a Mann-Whitney U test (P<0.05). **B**. The DENV-2 infection rate was determined by an indirect fluorescent antibody assay performed on head squashes of individual Waco and WB1 females of *Ae. aegypti* at 14 and 21 days (n = 36) or of Houston and HT1 females of *Ae. albopictus* at 14 days post-infection (n = 30). Data shown are means of three replicates for Waco and WB1 and two replicates for Houston and HT1, and the bars indicate standard error. There was a significant difference in the infection rate between the Waco and WB1 strains at both 14 and 21 days post-infection (Fisher's exact test, p<0.05).

We also compared the dissemination of DENV-2 within the WB1 and Waco strains of *Ae. aegypti* by measuring the number of copies of the dengue genome in the mosquito thorax. From Day 3 to Day 9, we saw no significant difference in the level and prevalence of dengue infection between the WB1 and Waco strains. A very low level of infection was detected in one of five replicates in both strains on Day 6. On Day 9, all five Waco replicates and four of the five WB1 replicates were positive for dengue infection. Whereas DENV-2 accumulated in the thorax of the Waco strain mosquitoes from Day 12 to 18, a strong *Wolbachia*-mediated inhibition effect was observed in the WB1 mosquitoes. On Day 12, the amount of DENV-2 in the thoraces of the WB1 mosquitoes was 2.6×10^5^ times lower than in the Waco strain (Mann-Whitney U test, P<0.05) (Figure 2A). On Days 15 and 18, three of five WB1 replicates had no detectable dengue infection, whereas all the Waco thoraces were heavily infected by DENV.

Similar results were obtained when we used IFA to assay female heads. Only 38.9% of the mosquitoes were positive for the DENV-2 E protein in the heads of WB1 mosquitoes by Day 14, as compared to 94.4% of the Waco mosquitoes (Fisher's exact test, p<0.05). By Day 21, 91.7% of the Waco strain mosquitoes were positive for DENV in the head squash assay, whereas only 5.6% of the WB1 strain mosquitoes were positive (Fisher's exact test, p<0.05) (Figure 2B). These results indicate that inhibition of DENV dissemination in the mosquito increases with time.

Experiments were also conducted to examine whether a similar *Wolbachia*-mediated inhibition of DENV could occur in *Ae. albopictus*. After both *Wolbachia*-infected and uninfected *Ae. albopictus* were fed on the same infectious blood, 100% of the mosquitoes were positive in the head squash assay in both groups (Figure 2B). Thus, *Wolbachia*-mediated inhibition of DENV was not observed in *Ae. albopictus*, at least not when this particular viral titer of the infectious blood was used to infect the mosquitoes.

### *Wolbachia*-mediated reduction of the DENV transmission potential of *Ae. aegypti*

**Figure 3:**
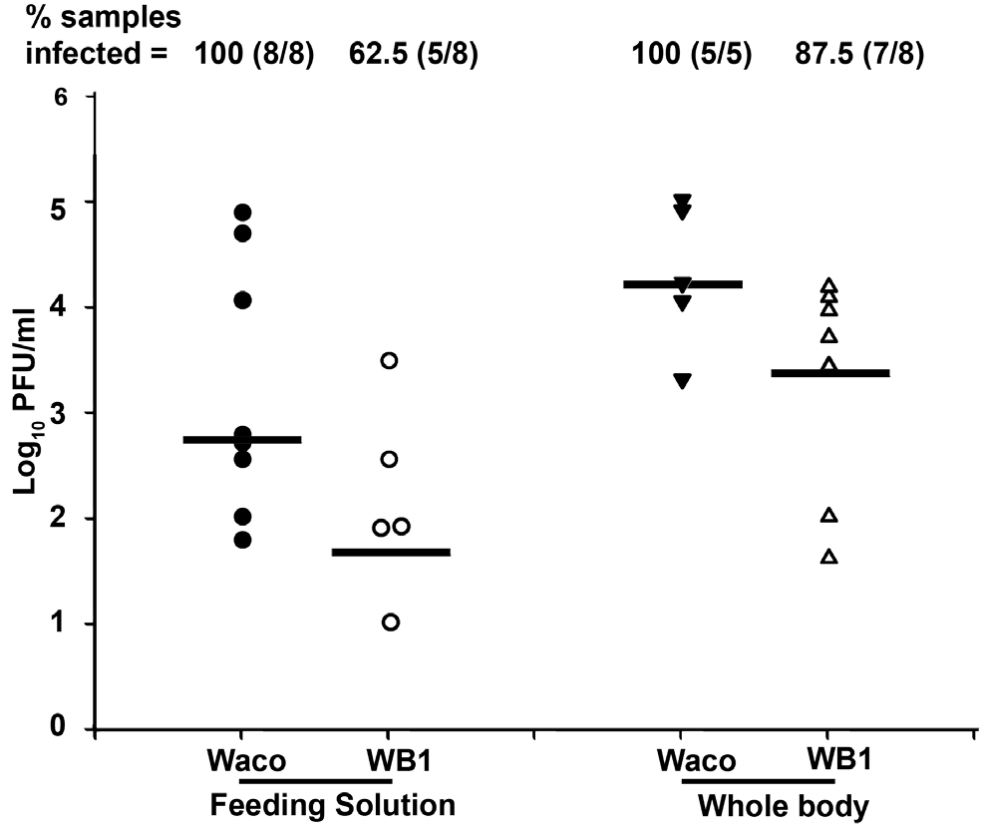
*In vitro* assay of DENV-2 transmission by Waco and WB1 mosquitoes at 14 days post-infection. After the wings and legs had been removed, the proboscis of each mosquito was inserted into a feeding solution for 90 min. Solutions from eight mosquitoes were pooled as one group and analyzed for infectious DENV-2 by plaque assays. The viral titers of each group of mosquitoes after feeding on the solutions were also analyzed in parallel. Lines indicate the log of the median values from five or eight biological replicates. Viral titers were significantly higher in the Waco strain than the WB1 strain for both the feeding solution and whole body (Mann-Whitney U test, P<0.05).

To determine whether mosquitoes' potential to transmit DENV-2 was inhibited by *Wolbachia*, we compared the levels of virus particles released during feeding from the proboscis of WB1 and Waco strain mosquitoes. At 14 days post-infection, the mosquitoes were allowed to feed on an artificial feeding solution for 90 min, and the viral titers in the solution were then measured by plaque assay. The median titer of viruses released from the mosquito proboscis was 12 times higher in the Waco strain (5.5×10^2^ pfu/ml) than in the WB1 strain (45 pfu/ml) (Mann-Whitney U test, P<0.05) (Figure 3). The virus infection rate of the feeding solution from the Waco strain was also significantly higher than that from the WB1 strain. Of the eight groups of pooled feeding solution from WB1 mosquitoes, three had no titer of virus at all, and one had a titer of 10 pfu/ml. Because each feeding group consisted of eight female mosquitoes, this result indicates that *Wolbachia* can completely block dengue transmission potential in at least 37.5% (24 of 64) of WB1 mosquitoes. In contrast, positive titers were detected in all the eight groups of pooled feeding solution from the Waco strain, and three groups had viral titers of 1.1×10^4^ to 7.6×10^4^ pfu/ml. These observations were consistent with the results from trituration of the whole bodies from each mosquito group: The median titer of the five Waco groups was 1.6×10^4^ pfu/ml virus, as compared to 2.5×10^3^ pfu/ml virus for the eight WB1 groups. No virus was detected in one of eight WB1 whole-body groups (Figure 3). These results suggest that the DENV-2 transmission potential had been greatly reduced by *Wolbachia* in *Ae. aegypti*.

In order to allow us to correlate the data between different assays, we compared the dengue infection levels in the Waco and WB1 strains at 7 days post-infection, in parallel with the results of the qRT-PCR and plaque assays. Although both the assays showed a significant reduction in the viral infection in the midguts and whole bodies, qRT-PCR showed a higher -fold reduction than the plaque assay in both tissues (Table S1). In particular, the virus infection in whole bodies and midguts were reduced by 2.4×10^4^- and 100-fold according to the qRT-PCR results, but only by 139.4- and 10.6-fold, respectively, when measured by plaque assay. Moreover, comparison of the inhibition in midguts and whole bodies also showed a difference, with whole bodies exhibiting stronger inhibition than midguts at 7 days post-infection (Table S1).

### *Wolbachia* tissue distribution, density and infection frequency in the WB1 strain

**Figure 4:**
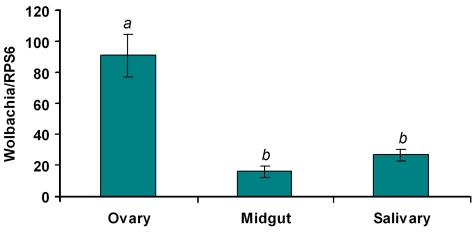
*Wolbachia* density in the ovaries, midguts and salivary glands of WB1 mosquitoes. Total RNA was extracted from one ovary or midgut or a pool of three salivary glands using the RNAeasy kit. q-PCR was conducted using primers targeting the *w*AlbB-wsp gene. The *Wolbachia* genome copy number was normalized with *Ae. aegypti* RPS6. Two recombinant plasmids containing the targeted fragments were used to generate separate standard curves for *w*AlbB-wsp and RPS6. Ten biological replicates were used for each tissue. Error bars represent the standard error; different letters are significantly different (ANOVA, P<0.05).

*Wolbachia*-mediated viral inhibition might be related to the tissue distribution, density and infection frequency of *Wolbachia* in WB1 mosquitoes. In our analysis of these aspects of *Wolbachia* infection, we focused on midguts and salivary glands, two important tissues targeted by DENV. The number of copies of *Wolbachia* genomic RNA in the two tissues was measured by quantitative PCR and compared to that in the ovaries. *Wolbachia* was present in both midguts and salivary glands at a 3- to 5-fold lower density than in ovaries: Specifically, there were 16.5 and 27.2 copies of the *Wolbachia* genome (normalized by the ribosomal protein S6 [RPS6] copy number) in the midguts and salivary glands, respectively, as compared to 91.5 copies in the ovaries (Figure 4). In order to confirm that each individual WB1 mosquito was carrying the *Wolbachia* bacterium, we randomly selected 15 females from the current WB1 population cage and tested their infection status by PCR. As had been observed 6 years ago [6], all of the 15 were positive for *Wolbachia*.

### Up-regulation of Toll pathway genes in *Ae. aegypti* by *Wolbachia*

**Figure 5:**
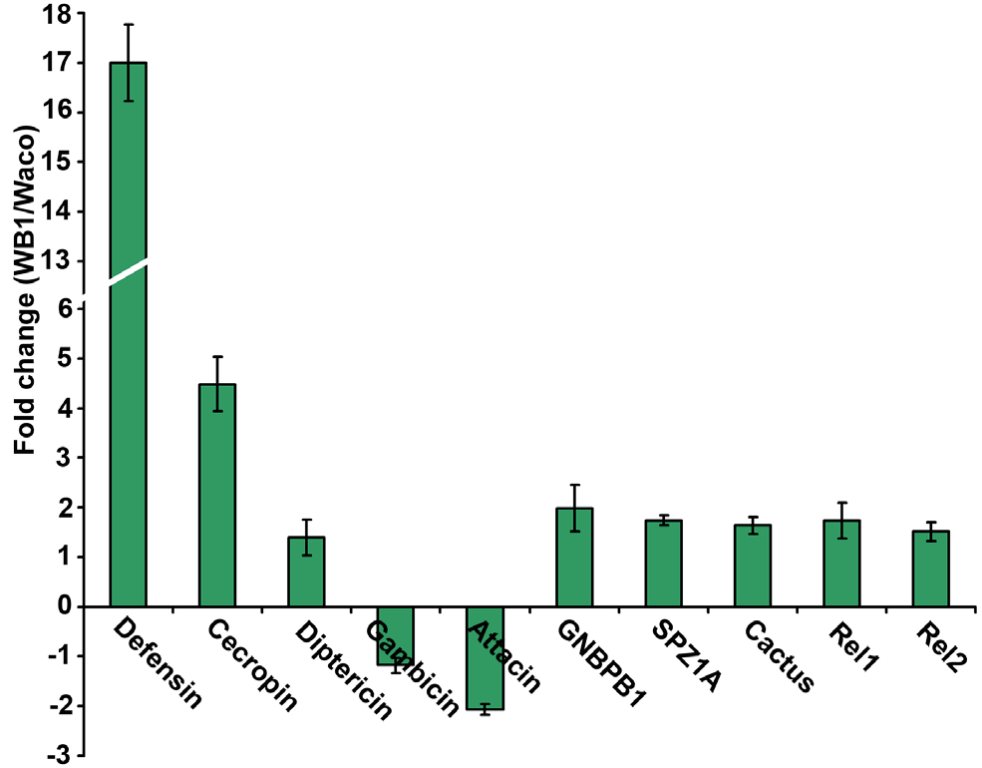
Expression of selected immune genes induced by *Wolbachia* in Waco and WB1 mosquitoes. qRT-PCR was performed using whole bodies of 4- or 5-day-old non-blood-fed females, with three biological replicates for each gene. Gene expression data were normalized with RPS7. The primer sequences have been reported previously [14]. The bars indicate standard error.

The results of our previous studies have suggested that microbial flora in the mosquito might mediate the anti-dengue response by boosting the mosquito's basal immunity [14]. We therefore investigated the possibility that *Wolbachia* can elevate basal immunity in *Ae. aegypti*. By comparing the expression of selected immune genes in 4- or 5-day-old non-blood-fed females of the Waco and WB1 strains, we found that a number of immune genes were up-regulated by *Wolbachia* (Figure 5). Specifically, *Wolbachia* induced a 17-fold increase in defensin expression and 4.49-fold increase in cecropin expression. Up-regulation was also observed for other Toll pathway genes, including Rel1, Spz1A and GNBPB1. These results indicate *Wolbachia* can activate the Toll pathway and boost basal level immunity in *Ae. aegypti*. Considering that the mosquito Toll pathway can control dengue infection in mosquitoes, this effect might represent a potential mechanism underlying the suppression of dengue infection by *Wolbachia*.

### *Wolbachia*-mediated increase in the longevity of dengue-infected *Ae. aegypti*

**Figure 6:**
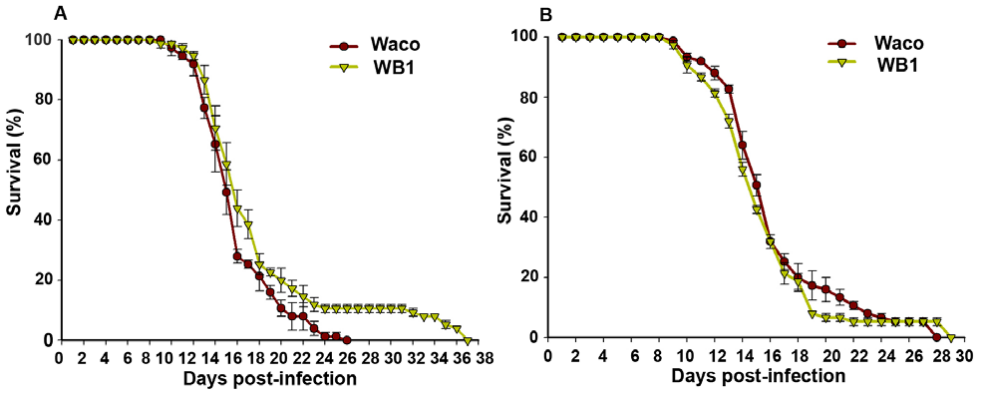
Longevity of Waco and WB1 mosquitoes fed with blood mixed with or without DENV. After mosquitoes were fed a blood meal, either containing DENV (A) or not (B), they were maintained in an incubator at 27°C and 85% humidity. The data shown are means of three replicates (25 for each), and the bars indicate standard error. Results from two independently reared cohorts are shown (cohort 1 [A]; cohort 2 [B]). The survival curves were significantly different between the Waco and WB1 mosquitoes fed with dengue-infected blood (logrank test, p<0.0001).

To determine whether suppression of DENV-2 infection by *Wolbachia* provides any benefit to the mosquito, we compared the relative survival of the Waco and WB1 strains after infection with DENV-2. WB1 females lived significantly longer than Waco females (logrank test, P<0.05) (Figure 6A). This difference was manifested at a late stage, with all of the Waco females having died by 26 days post-infection, but 10% of the WB1 females living for up to 12 additional days. This result produced a “long tail” effect in the survivorship distribution. To determine whether this increase in WB1 survival was specifically associated with DENV-2 infection, we fed Waco and WB1 mosquitoes with blood lacking DENV. Under these conditions, we observed no difference in survival between the two strains (Figure 6B). These results indicated that *Wolbachia* can slightly increase the longevity of WB1 only when the mosquitoes are infected with DENV.

## Discussion

*Ae. aegypti* is not naturally infected by *Wolbachia*, but a transfected line WB1 was previously successfully developed in the laboratory. This WB1 line was able to invade the wild-type laboratory population; population replacement can occur in seven generations after the initial release [6]. Although *Wolbachia* is widely used as a driver to spread disease-resistant genes into the mosquito, the impact of *Wolbachia* itself on pathogens has not been well understood. Given the large overlap in the tissue distribution and intracellular localization of *Wolbachia* and DENV in mosquitoes, we conducted experiments to characterize the interactions between *Wolbachia* and DENV. Our results show that *Wolbachia* inhibits viral replication, dissemination and transmission in mosquitoes. This inhibitory effect was associated with an elevated level of basal immunity in the mosquitoes.

We found that the inhibition of DENV infection in *Wolbachia*-infected WB1 mosquitoes occurred in a variety of tissues. This observation might reflect the broad tissue distribution of *w*AlbB in the WB1 strain, especially in those tissues in which DENV replicates and resides within the mosquito. DENV enters the mosquito midgut epithelial cells following an infectious bloodmeal, with the infection reaching a peak in the midgut from 7 to 10 days post-infection, in the salivary glands from 10 to 17 days post-infection, and in the head after 14 days post-infection [8]. We confirmed the presence of *Wolbachia* in the midguts and salivary glands as well as the ovaries of WB1 mosquitoes. Although the amount of *Wolbachia* in ovaries was three to five times higher than that in the midguts and salivary glands, a significant level of *Wolbachia* was also present in those two tissues. This distribution pattern of *w*AlbB was consistent with what was observed in the original host, *Ae. albopictus*, in which *Wolbachia* is widely distributed throughout the host tissues, both reproductive (e.g., ovaries and testes) and non-reproductive (e.g., hemolymph, midgut, muscle, wing and head) [11].

In our study, the viral inhibitory effects mediated by *Wolbachia* were consistently observed in different assays, at different time points and in different tissues. At 14 days post-infection, all the Waco strain feeding solutions contained DENV, but at least 37.5% of the WB1 feeding solutions had no DENV, as determined by plaque assay. Similarly, the DENV genome could not be detected by qRT-PCR in 40% (6/15) of the WB1 midguts or 60% (9/15) of the WB1 thoraces at 15 days post-infection; 61.1% of the WB1 mosquito heads were also negative for the DENV-2 E protein, as detected by IFA at 14 days post-infection. However, it appeared that *Wolbachia* differentially affects the replication of DENV and the formation of infectious virions, since the inhibitory effect in both the midguts and whole bodies at 7 days post-infection was higher when measured by qRT-PCR than when measured by plaque assay.

Our results also indicated that the level of inhibition was different in different tissues and at different time points. This variation might be related to the dynamics of DENV and the distribution of *Wolbachia* in mosquitoes. For example, *Wolbachia* might mediate a stronger viral inhibition in a tissue that contains a higher amount of *Wolbachia* but a low level of DENV than in another tissue that contains a low amount of *Wolbachia* but a high level of DENV. Although a significant reduction in dengue infection was observed on Days 3, 6, 12 and 15 in the midguts of WB1 mosquitoes, the number of viral genome copies on Days 3 to 9 was not significantly different in the thoraces of WB1 mosquitoes than in those of Waco mosquitoes. In contrast, only a trend toward a reduction was observed, perhaps because most of the DENV had not yet escaped from the midgut during this period. The very low number of virus particles present there made it difficult to distinguish between signal and noise in the assay.

Inhibition of DENV might be caused by the activation of certain host defense responses as a result of *Wolbachia* infection. Virus-inhibitory effects have been observed in human infected with a close relative of *Wolbachia*, *Orientia tsutsugamushi*; these effects appear to be caused by binding to the virus of antibodies against bacteria [16]. In arthropods, innate immunity plays an important role in limiting pathogen infection. Such immune responses are largely regulated by two main pathways, the Toll and Imd pathways [17],[18]. In *Drosophila*, the Toll pathway is mainly involved in defense against fungi and Gram-positive bacteria, while the Imd pathway affects resistance to Gram-negative bacteria. In response to either the *Drosophila* X virus (a member of the *Birnaviridae*) or *E. coli* infection, *D. melanogaster* induces the same antimicrobial peptide genes. This commonality suggests that these two diverse classes of pathogen can activate the same immune response pathway in the insect host [19]. More importantly, we have recently found that activation of the mosquito Toll pathway can suppress DENV infection [14]. A recent genome-wide analysis of the *Wolbachia*-host interaction has revealed that *Wolbachia* infection has an impact on a broad range of physiological systems in the host, including innate immunity [15].In the present study, we also observed an elevated mosquito immune response in the WB1 strain. Thus, the observed inhibition of dengue infection in the WB1 strain may be partially explained by a *Wolbachia*-induced up-regulation of Toll pathway genes.

Alternatively, *Wolbachia*-mediated viral interference could also be the result of a direct competition between DENV and *Wolbachia* for the same resources, or of an indirect perturbation by *Wolbachia* of the cellular environment required by DENV. As a parasite, DENV depends on the metabolic network of the host cell to provide the energy and macromolecular subunits necessary for its replication. By producing metabolic alterations in its host, *Wolbachia* may interfere with dengue replication.

It is unlikely that the inhibitory effects on DENV that we observed in the WB1 mosquitoes were caused by differences in genetic background between the WB1 and Waco strains. After the WB1 strain was initially produced from the Waco strain by embryo microinjection, 50 virgin WB1 females were out-crossed with 50 Waco males for six generations to homogenize their genetic background [6]. Since then, the population cages housing the Waco and WB1 strains have been maintained in the laboratory under identical environmental conditions.

While this manuscript was in review, an independent report with similar findings to ours was published: In agreement with our data, another type of *Wolbachia*, *popcorn*, was found to inhibit the ability of a range of pathogens, including DENV, Chikungunya, and *Plasmodium*, to infect *Ae. aegypti* [20]. This result indicates that *Wolbachia* may induce a general killing mechanism in the host or influence common host factors or networks that are required for a variety of parasites. It appears that such an effect occurs locally but not systematically, because DENV can be present in cells of *Wolbachia*-infected mosquitoes that lack *Wolbachia*, and the strength of the effect depends on the on-site density and the type of *Wolbachia*. In addition, in neither this published study nor our current study was a similar interference effect observed in *Ae. albopictus* or *Ae. fluviatillis*, in which *Wolbachia* infection occurs naturally. This apparent selectivity suggests that the observed interference may require a specific *Wolbachia*/host combination, or it may be associated with the recent establishment of *Wolbachia* in the host.

Our results suggest the DENV-2 transmission potential in *Ae. aegypti* can be strongly inhibited by concurrent infection with *Wolbachia*. At 14 days post-infection, *Wolbachia* in *Ae. aegypti* could completely block dengue transmission in at least 37.5% of the mosquitoes. This inhibitory effect appeared to become stronger over time. While more than 90% of the female heads were infected in the Waco strain at both 14 and 21 days post-infection, the infection rate dropped from 38.9% to 5.6% between those two days in the case of the WB1 strain. *Wolbachia*-induced inhibition of DENV infection provides us with a bonus for using this endosymbiont to block dengue transmission in the mosquito. The anti-dengue effect of this endosymbiont is not solely dependent on the effector transgenes carried by *Wolbachia*. Therefore, the main requirement for the effector transgenes is merely to eliminate any dengue viruses that survive the *Wolbachia*-induced suppression. This added advantage might enhance the efficiency of an anti-dengue effector, because a lesser dose of effector would be necessary when it is expressed by and delivered from *Wolbachia* than if it were to function alone. The anti-dengue effect of *Wolbachia* can also reduce concerns over losing the link between antipathogen and the transgene driver, because *Wolbachia* alone can confer resistance to DENV in mosquitoes. Also, *Wolbachia* may be able to be used directly to blocking dengue transmission without linkage to an anti-dengue gene. This strategy will, however, require a better understanding of the mechanism underlying the dengue inhibition effect conferred by *Wolbachia*. By improving the efficiency of this inhibition mechanism, a complete blockade of dengue transmission could potentially be achieved. Finally, although DENV was still present in the proboscis of some WB1 mosquitoes in our study, the titer was 12 times lower than in the Waco strain. It will be interesting to know whether this level is below the threshold viral titer that is required to cause infection in humans.

As has been reported in *Drosophila* [12],[13], we observed that *w*AlbB could increase the survival rate of the dengue-infected mosquitoes. Such an increase in survival was not observed when WB1 were fed uninfected blood. However, in another study, when *Ae. albopictus* were fed with blood without DENV, *Wolbachia* (a superinfection of *w*AlbA and *w*AlbB, referred as *w*AlbA&B) was reported to provide a fitness advantage, including an increase in the mosquitoes' longevity [21]. This difference in the observed results might reflect differences in experimental design, infection status, or mosquito species. Thus far, *Wolbachia* has been reported to affect the life span of its mosquito hosts in two different directions, with the longevity reduced or increased by *w*MelPop or *w*AlbA&B, respectively [9],[10],[21]. Although the underlying mechanism is still unclear, it is possible that *Wolbachia* interacts with certain biological pathways, such as the insulin signaling pathway [22], which can influence the host's life span.

It is still unknown whether this slight increase in survival that we observed for the infected mosquitoes could have a negative effect on disease control. To test this possibility, it will be necessary to determine whether this small fraction of older WB1 mosquitoes has cleared the viral infection, and whether these mosquitoes still have the ability to feed on their hosts. Moreover, the longevity of mosquitoes in the field, where the majority live fewer than 30 days, is quite different from that of mosquitoes reared under laboratory conditions. Future studies are needed to assess the overall impact on dengue transmission of this *w*AlbB-induced resistance to dengue infection and increase in life span.

In summary, we have demonstrated an inhibitory effect of *Wolbachia* on DENV in *Ae. aegypti*. This inhibition, which was found to occur in the midgut, thorax and head, further reduced the DENV transmission potential of the mosquitoes. We have also provided evidence that the inhibitory effect may be related to an elevated basal immunity produced by the *Wolbachia*. Our results provide support for future experiments to elucidate the mechanism underlying the inhibition of dengue infection by *Wolbachia* and to dissect the three-way interactions among DENV, *Wolbachia* and the mosquito. From an application standpoint, we have demonstrated that *Wolbachia* can be used not only as a transgene driver but also as an effector to suppress dengue infection in the mosquito *Ae. aegypti*. When compared to other potential driver systems, the effector function of *Wolbachia* offers an additional advantage and facilitates its implementation as a means of blocking dengue transmission by mosquitoes.

## Materials and Methods

### Mosquito rearing and cell culture maintenance

All the mosquito strains used in these experiments, including the wild-type Waco strain and the transfected line WB1 of *Ae. aegypti*, the wild-type Houston strain and tetracycline-treated HT1 strain of *Ae. albopictus*, were maintained on sugar solution at 27°C and 85% humidity with a 12-hr light/dark cycle according to standard rearing procedures. The *Ae. albopictus* cell line C6/36 was grown in minimal essential medium (MEM) with 10% heat-inactivated FBS, 1% L-glutamine, and 1% non-essential amino acids at 32°C and 5% CO_2_.

### DENV-2 infections

The New Guinea C strain of DENV-2 was propagated in C6/36 cells according to standard conditions [23]: In brief, 0.5-ml aliquots of virus stock were used to infect 75-cm^2^ flasks of C6/36 cells, at 80% confluence, with a multiplicity of infection (MOI) of 3.5 virus particles/cell. Infected cells were incubated for 10 days, and the medium was changed on Day 5. Cells were harvested with a cell scraper and lysed by repeated freezing and thawing in dry ice and a 37°C water bath. The resulting virus suspension was mixed 1∶1 with commercial human blood. A flask with uninfected C6/36 cells was maintained under similar conditions and used to create the noninfectious blood meal that served as our control. The blood meal was maintained at 37°C for 30 min prior to use for feeding 7-day-old mosquitoes (http://www.jove.com/index/Details.stp?ID=220).

### Mosquito dissections

Mosquitoes at 3, 6, 9, 12 and 15 days post-infection were dissected to collect the midguts and thorax in RNALater, with three individual mosquitoes in a single replicate. Five replicate biological assays were performed. Total RNA was extracted using the RNeasy kit (QIAGEN). To measure the virus titers in mosquito bodies, at 14 days after a blood meal, mosquitoes were briefly washed in 70% ethanol, then rinsed in sterile distilled water. The midgut and thorax were dissected in sterile PBS and transferred separately to microcentrifuge tubes containing 150 µl of MEM, then homogenized with a Kontes pellet pestle motor in a sterile environment.

### Real-time qPCR assays

To measure the number of viral genome copies, total virus RNA was extracted using the RNeasy kit (QIAGEN) and reverse-transcribed using Superscript III (Invitrogen, Carlsbad, California, USA) with random hexamers. qRT-PCR was conducted using primers targeting the dengue NS5 gene and the host RPS6 [24]. The dengue genome copy number was normalized using the RPS6 results. Two recombinant plasmids containing the targeted fragments were diluted from 10^1^ to 10^8^ copies/reaction and used to generate separate standard curves for NS5 and RPS6. Real-time quantitation was performed using the QuantiTect SYBR Green PCR Kit (Qiagen) and ABI Detection System ABI Prism 7000 (Applied Biosystems, Foster City, California, USA). Three independent biological replicates were assayed, and all PCR reactions were performed in triplicate. To determine the number of copies of the *Wolbachia* genome and assess the expression of mosquito immune genes, real-time PCR was carried out as previously described [14],[25].

### Plaque assays for DENV-2 virus titration

Virus titers in the tissue homogenates were measured as previously reported (http://www.jove.com/index/Details.stp?ID=220): The virus-containing homogenates were serially diluted and inoculated into C6/36 cells in 24-well plates. After incubation for 5 days at 32°C and 5% CO_2,_ the plates were assayed for plaque formation by peroxidase immunostaining, using mouse hyperimmune ascitic fluid (MHIAF, specific for DENV-2) and a goat anti-mouse HRP conjugate as the primary and secondary antibodies, respectively.

### Indirect immunofluorescence assay (IFA)

On Days 14 and 21 after infection, the viral antigen in the heads of mosquitoes was detected by using an indirect IFA. Mosquito heads were cut off from the thorax with a razor blade, transferred to a pre-cleaned glass microscope slide, and then squashed under a cover slip. After being air-dried for 10 min at room temperature, the slides were fixed in cold acetone (−20°C) for 10 min and then dried. A mouse anti-dengue complex monoclonal antibody (Millipore) and a fluorescein-conjugated affinity-purified secondary antibody (Millipore) were used in all head squash assays. Specimens were examined with a Zeiss (Germany) fluorescence microscope. For the samples that yielded ambiguous results in the IFA assay, RT-PCR was conducted in parallel to confirm the infection status.

### Longevity assay

Adult female 1-week-old mosquitoes were fed with either blood alone or with blood mixed with DENV-2, as described in the section on DENV-2 infections. After 30 min of feeding, the engorged females were sorted on the Carbon Dioxide Staging, transferred to cardboard containers and then incubated at 27°C with 80% humidity while being fed a 10% sucrose solution. All containers were checked for deaths daily, and the surviving mosquitoes were transferred to a new clean container every week until all the mosquitoes died. The parameter used to measure lifespan was the mean of the survival percentages for three biological replicates of 25 mosquitoes each.

### Transmission assay

Mosquitoes that had been infected with DENV-2 as described above were maintained for 14 or 21 days for forced salivation assays. The assays were conducted as previously reported [2],[26]: In brief, mosquitoes were deprived of food for 24 h prior to forced salivation. The legs and wings of each mosquito were cut away, and the proboscis was inserted into 25 µl of feeding solution (50% FBS/164 mM NaCl/100 mM NaHCO_3_/0.2 mM ATP/≈50 µg sucrose/phenol red, pH 7.0) [2] in a 0.2-ml PCR tube. After 90 min, the mosquitoes were removed, and the feeding solutions from eight mosquitoes (one group) were combined and sterilized by Millex-GV filter for plaque assays. Whole bodies of eight mosquitoes from the same group were homogenized in 350 µl MEM. After filtration, the supernatant was used for plaque assay. Eight biological replicates were used for each treatment.

### Accession numbers

The Entrez Gene IDs for the genes and proteins mentioned in the text are 5565922 (Cactus), 5569526 (REL1A), 5578608 (Caspar), 5569427 (REL2), 5579094 (DEF), 5579377 (CEC), 5578028 (Attacin), 5565542 (Diptericin), 5579192 (GNBPB1) and 5564993 (Gambicin).

## Supporting Information

Table S1 Comparison of the *Wolbachia*-mediated inhibitory effect on DENVs as measured by qRT-PCR and plaque assay. The virus infection was measured in parallel by the two assays in midguts and whole bodies at 7 days post-infection. Six or five biological replicates were used in plaque assay or qRT-PCR, respectively. Data are shown as the median -fold reduction.
(0.04 MB DOC)
