# Supplementary material for: The Endosymbiotic Bacterium Wolbachia Induces Resistance to Dengue Virus in Aedes aegypti
Source: PLoS Pathog. 2010 Apr 1;6(4):e1000833. doi: 10.1371/journal.ppat.1000833 (PMC2848556; doi:10.1371/journal.ppat.1000833)
Supplement: Table S1 — Comparison of the Wolbachia-mediated inhibitory effect on DENVs as measured by qRT-PCR and plaque assay. The virus infection was measured in parallel by the two assays in midguts and whole bodies at 7 days post-infection. Six or five biological replicates were used in plaque assay or qRT-PCR, respectively. Data are shown as the median -fold reduction. (0.04 MB DOC) [file ppat.1000833.s001.doc]

**Table S1.** **Comparison of the *Wolbachia*-mediated inhibitory effect on DENVs as measured by qRT-PCR and plaque assay**.

|  | **qRT-PCR** | **Plaque assay** |
| --- | --- | --- |
| **Midgut** | 100.0 | 10.6 |
| **Whole body** | 2.4 x 10^4^ | 139.4 |

The virus infection was measured in parallel by the two assays in midguts and whole bodies at 7 days post-infection. Six or five biological replicates were used in plaque assay or qRT-PCR, respectively. Data are shown as the median -fold reduction.
